# The role of magnetic resonance imaging (MRI) in invasive lobular breast cancer based on mammographic density

**DOI:** 10.1186/1470-7330-15-S1-P20

**Published:** 2015-10-02

**Authors:** GJ Bansal, D Santosh, E Davies

**Affiliations:** 1 Breast Centre Llandough, University Hospital of Llandough, Penlan Road, Llandough, CF64 2XX, UK

## Patients and methods

We carried out a single centre retrospective analysis of all lobular cancers diagnosed between 2011-2015. We divided the patients into two groups, one with MRI performed preoperatively and other with no MRI. We analysed mammographic density, imaging size and post-operative histological size between the two groups. We also compared their surgical procedures and analysed if surgical procedure was altered after MRI. In case of alteration, we analysed if the change was adequate by comparing post-operative histological findings.

## Results

There were a total of 97 patients, 36 had pre-operative MRI and 61 had no MRI. 27/36 (75%) in the MRI group had mammographic density > 50% versus 17/61 (27.8%) in the ‘no MRI’ group (p=0.009). MRI picked clinically relevant findings in 22/36 (61.1%) of patients.12/36 (33.3%) had mastectomy following MRI, out of which 9 (25%) had change in surgical therapy following MRI. There was no overtreatment in the MRI group. Patients in the ‘No MRI’ group had larger histological size of tumours following mastectomy, with a higher mastectomy rate 26/61(42.6%) in this group, which was again appropriate.

## Conclusion

Our choice of MR in preoperative planning of invasive lobular patients, based on mammographic density seems adequate. MRI led to appropriate change in surgical therapy in 25%. Although other factors such as patient choice for either mastectomy or conservative treatment plays an important role in pre-operative planning, we recommend breast mammographic density as one of the criteria.

